# Risk stratification models in human papillomavirus-associated oropharyngeal squamous cell carcinoma: the Nova Scotia distribution

**DOI:** 10.1186/s40463-019-0325-z

**Published:** 2019-01-14

**Authors:** David Forner, Matthew H. Rigby, Derek Wilke, S. Mark Taylor, Nathan Lamond

**Affiliations:** 1 Division of Otolaryngology – Head & Neck Surgery, Department of Surgery, Dalhousie University, Halifax, Nova Scotia Canada; 2 Department of Radiation Oncology, Dalhousie, Halifax, Nova Scotia Canada; 3 Division of Medical Oncology, Department of Internal Medicine, QEII - Bethune Building, Dalhousie University, Suite 470, 1276 South Park Street, Halifax, Nova Scotia B3H 2Y9 Canada

## Abstract

**Objective:**

The incidence of oropharyngeal squamous cell carcinoma is increasing with a growing proportion of diagnoses associated with human papillomavirus (p16 + OSCC), which generally confers a favorable prognosis. For these reasons, novel risk stratification models specific to the p16 + OSCC population have recently been proposed to guide future research on treatment de-intensification for appropriate patients.

This study aimed to quantify patient risk distribution using multiple published risk models and investigate the hypothesis that the local p16 + OSCC population includes a smaller proportion of low-risk patients due to a high prevalence of concurrent tobacco exposure.

**Methods:**

A retrospective cohort study was performed including patients diagnosed with p16 + OSCC in Nova Scotia between 2011 and 2015. Patient identification was obtained through the CCNS registry and an institutional database. Exclusion criteria included HPV negative status, second primary cases, incomplete data availability, and local recurrence cases.

**Results:**

Following exclusion, 117 patients met study criteria. The majority had small primary tumors (70.9% ≤ T2) and advanced nodal status on presentation (60.7% ≥ N2b). Most patients had a positive smoking history (62.4%), with 53.0% of patients having a pack-year history greater than 10 pack-years. In four of the five risk stratification models, the majority of the study population fell into the lowest risk category. The risk stratification distribution of our local population was similar to the populations used to validate the published models, with the largest single category difference being 13.3% (range − 12.3 to + 13.3%).

**Conclusions:**

This is the first study to compare multiple currently published risk stratification models to a local population and address the uncertainty of risk stratification in the Nova Scotian p16 + OSCC population. Despite a high prevalence of concurrent tobacco exposure, the study population was found to be overall low risk, with similar risk compared to model validation populations.

## Introduction

Oropharyngeal squamous cell carcinoma (OSCC) accounts for over 1300 new cancer diagnoses in Canada each year [1]. OSCC has traditionally been associated with alcohol and tobacco use, accounting for up to 75% of cases [2], but public health initiatives have resulted in an overall decline in head and neck cancers. Despite this, rates of OSCC have continued to rise [3, 4]. This trend is due to a growing proportion of cases associated with infection by the human papillomavirus (HPV) [4]. In Canada, the proportion of HPV associated cases has risen to approximately 75% [5]. Immunohistochemistry for the expression of p16 has been correlated to the presence of HPV associated disease, and therefore p16 positivity was considered to be HPV association in this study.

HPV-associated OSCC (p16 + OSCC) appears to be a unique disease entity from non-HPV associated OSCC. p16 + OSCC is associated with a more favourable prognosis in general, with improved rates of locoregional control and survival, compared to non HPV-associated OSCC (p16-OSCC) [6]. Current management approaches in patients diagnosed with HPV-OSCC often employ multimodality treatment including combinations of surgery, radiotherapy, and chemotherapy. Unfortunately, these treatments are associated with significant long-term toxicity, including organ sacrifice, severe dysphagia, and speech impairment [7].

The combination of superior cancer outcomes in the p16 + OSCC patient population as well as its younger age at the time of diagnosis [8] means that more head and neck cancer patients are now living with chronic toxicities from treatment. This has prompted interest in the possibility of treatment de-escalation for appropriate patients in an effort to improve toxicities without compromising cancer outcomes.

Ongoing clinical trials examining a variety of de-intensification approaches suggest that low-risk patients may have progression free survival and overall survival above 95% [9].

In order for p16 + OSCC patients to benefit from treatment deintensification, treatment must be guided by accurate prognostic models. Recently, several key factors affecting patient prognosis have been identified [10] and putative risk stratification models have been developed [10–13]. Smoking, lymph node involvement, age, and patient comorbidities play an integral role in this determination, as anatomic staging alone has been deemed insufficient for prognostic purposes in p16 + OSCC [14]. The American Joint Committee on Cancer (AJCC) has therefore significantly altered its staging system for oropharyngeal cancers, creating a separate staging system for HPV-associated disease in its most recent edition [15].

Little is known about the p16 + OSCC population in Nova Scotia, and information regarding risk distribution in these patients is similarly lacking. Nova Scotia has a high prevalence of smoking, behind only the Territories as the province with the highest smoking rates in Canada [16]. This study aimed to quantify patient risk distribution using multiple published risk models and investigate the hypothesis that the local p16 + OSCC population includes a smaller proportion of low-risk patients due to a high prevalence of concurrent tobacco exposure. Through this objective, we also aimed to answer important questions regarding our local patient population, including the average age, sex distribution, and smoking rate of p16 + OSCC patients. This is the first study to directly compare a local study population to multiple putative risk stratification models, and the first study to identify the risk distribution in Nova Scotia.

## Methods

### Study design

This study was a retrospective cohort analysis of patients diagnosed with oropharyngeal squamous cell carcinoma between 2011 and 2015, inclusive. Patient data was obtained through the Cancer Care Nova Scotia patient registry and an institutional transoral laser microsurgery database. Electronic patient charts were subsequently reviewed for completeness. Patients were considered to have HPV-associated disease when they displayed p16 positivity on immunohistochemistry, defined as greater than 70% staining. Variables examined included gender, age, tumor-node-metastasis (TNM) staging, subsite of primary tumor, smoking history, and comorbidities at time of diagnosis. When available, pathological staging was used. When unavailable, clinical staging was utilized. American Joint Committee on Cancer Seventh Edition was utilized for TNM staging purposes during this study. Notably, following completion of data gathering in this study, the AJCC eighth edition has been released and adopted by our institution.

Smoking history and comorbidities were obtained from consultation notes, clinic follow-up notes, anesthesia assessments, and clinic nursing assessments. Patients were considered to have a positive smoking history when pack-year was greater than one. This is similar to Ang et al. which divided patients into never smoked, former smoker, and current smoker categories. Subsite of the primary tumor was determined by a physical examination performed by the treating head and neck surgeon, radiation oncologist, or medical oncologist.

Exclusion criteria included incomplete data availability, local recurrence cases, and second primary cases.

### Ethics approval

Ethics approval was granted by the Nova Scotia Health Authority Research Ethics Board.

### Models and analysis

Patients were stratified using previously reported stratification algorithms [10–13]. O’Sullivan and colleagues proposed a model using only HPV status, and tumor and nodal staging. Patients were stratified into non-HPV-OSCC and HPV-OSCC low and high risk in the original study. For the purposes of this study, the original stratifications were recalculated for p16 + OSCC only. Dahlstrom and colleagues likewise used tumor and nodal staging. The intermediate group was combined for calculation purposes. Ang and colleagues utilized HPV status, smoking status, and nodal status. Similar to O’Sullivan and colleagues, stratification was recalculated to include only p16 + OSCC. Finally, Huang et al. combined age, tumor and nodal staging, and smoking history to create four risk groups. The two intermediate groups were combined into a single intermediate group for this study.

Statistics were descriptive in nature and completed using SPSS Statistics (SPSS®, IBM®, version 21).

## Results

### Patient demographics

In total, 142 p16 positive patients were identified. Nine patients were excluded due to incomplete access to electronic medical records, and sixteen were excluded due to incomplete smoking status data. Therefore, 117 patients were included in the analysis.

The majority of patients were male (Table 1, 87.2%). The mean age at time of diagnosis was 58.1 years (Table 1, range 33–83). The majority of patients had T2 primary tumors or smaller on presentation (Table 1, 70.9%). The majority of patients had advanced nodal status on presentation, with 60.7% of patients presenting with N2b or greater. Of these patients, the greatest proportion was N2b, representing 41.9% of patients (Table 1).

**Table 1:** Patient demographics and TNM Staging

Variable	Number of Patients (%) or mean (range)
Male Gender	102 (87.2)
Age	58.1 (33–83)
Age > 70	6 (5.1)
Positive Smoking History	73 (62.4)
Pack-Year History	28.8 (2–150)
Continued Smoking	27 (23.1)
Cessation ≥10 y	26 (22.2)
Primary Tumor Subsite	
Base of Tongue	36 (30.8)
Tonsil	79 (67.5)
Vallecula	1 (0.9)
Soft Palate	1 (0.9)
Tumor Stage (T)	
T1	31 (26.5)
T2	52 (44.4)
T3	21 (17.9)
T4	12 (11.2)
Nodal Stage (N)	
N0	11 (9.4)
N1	13 (11.1)
N2a	22(18.8)
N2b	49 (41.9)
N2c	19 (16.2)
N3	3 (2.6)
Metastasis Stage (M)	
M0	117 (100)
M1	0 (0)

More than half of the patients had a positive smoking history (Table 1, 62.4%). Of the patients with a positive smoking history, the mean pack-year history was 28.8 pack-years (Table 1, range 2–150). Sixty-two patients had a pack-year history greater than 10 pack-years (53.0%), and 38 patients had a pack-year history greater than 20 pack-years (32.5%). Stratifying by gender showed 40% of females had a positive smoking history, with a mean pack-year history of 39.7 pack-years. Amongst men, both the prevalence tobacco exposure (37.3%) and mean pack-year history (27.3 pack-years) were lower.

### Risk stratification

Risk stratification using the model proposed by Ang et al. [10] revealed 66.7% of patients were low risk (Table 2). Age and pack-year history greater than 20 were combined with TNM staging by Huang and colleagues [12], and revealed no patients in the highest risk category in the local study population (Table 2). The majority of patients were in the lowest risk category (62.4%). The remaining patients were split between the middle risk categories.

**Table 2:** Risk distribution of local population compared to study population by stratification model

Model	Smoking History	Risk Level	Risk Description	Study Population	Model Population
Ang et al. 2010 [10]	HPV -ve: 73% HPV +ve: 65%	Low Risk	≦ 10 PYHx or > 10 PYHx with N0-N2a	66.7%	64.0%
		Intermediate Risk	> 10 PYHx with N2b-N3	33.3%	36.0%
O’Sullivan et al. 2013 [13]	HPV -ve: 94% HPV +ve: 64%	Low risk	T1-T3, N0-N2c	87.2%	74.9%
		High risk	T4,N0-N2c or N3	12.8%	25.1%
Huang et al. 2015 [12]	HPV -ve: 15 PYHx HPV +ve: 40 PYHx	Low Risk	≦ 20 PYHx	62.4%	49.4%
		Intermediate Risk	> 20 PYHx or Age ≦ 70	37.6%	45.9%
		High Risk	Age > 70	0.0%	4.7%
Dahlstrom et al. 2016 [11]	HPV +ve: 82.5%	Low Risk	T1, N0-N2	24.8%	29.5%
		Intermediate Risk	T2, N0-N2 or T3 or N3	64.1%	59.0%
		High Risk	T4	11.1%	11.5%

HPV + ve = p16 positive oropharyngeal squamous cell carcinoma, −ve = negative, PYHx = Pack Year History

Using O’Sullivan et al.’s model, the local patient population was low risk 87.2% of the time (Table 2). This was primarily driven by small primary tumor size. Dahlstrom et al. utilized a similar model [11], and the local patient population was most commonly in an intermediate risk category (Table 2, 64.1%). Twenty-nine patients (Table 2, 24.8%) were low risk.

## Discussion

This study investigated the p16 + OSCC risk distribution in Nova Scotia using multiple described risk stratification models. Specifics of Nova Scotian p16 + OSCC risk distribution are lacking and this was the first study to address these knowledge gaps in a patient population hypothesized to be high risk due to the high prevalence of tobacco exposure in the provincial population. To our knowledge, this is the first study to directly compare multiple risk stratification models in a local study population.

The prevalence of smoking in Nova Scotia is amongst the highest in Canada, behind only the Territories [17]. In this study, p16 + OSCC patients in Nova Scotia were found to have substantially increased smoking prevalence compared to both the provincial and national averages. This was especially true of the female p16 + OSCC population, which showed a smoking prevalence double the Nova Scotia age-adjusted population average (60% vs 30%) [18]. The overall local study population smoking prevalence was similar to the prevalence found in the risk stratification model populations, which ranged from 49 to 72% [10, 11, 13]. Similarly, the average pack-year history of smokers was non-significantly increased in this study compared to the Canadian average (28.8 vs 25.1 pack-years, *p* > 0.05) [19]. This quantity of tobacco exposure was similar to that seen in the validation cohorts of the previously published stratification models in which the average pack-year history was between 12.2 to 38 pack years [10–12].

Despite the high prevalence and quantity of concurrent tobacco exposure, the study population had a similar risk distribution compared to populations in published stratification models. Our study revealed a slightly decreased local population risk compared to the original study by Ang and colleagues [10]. Nova Scotia patients had a higher likelihood of a ten pack-year history (53.0% vs 50.6%). Despite this, the overall proportion of low risk patients was high (66.7 vs 66.4%). This is most likely due to a smaller proportion of ten pack-year patients having advanced nodal stage (N2b or greater; 62.9% vs 71.1%). The mean age was higher by about five years in our local population.

The model proposed by O’Sullivan and colleagues [13] yielded the greatest difference in stratification of all models examined. Nodal status was more favourable in the local study population compared to the population studied by O’Sullivan in which 69% of patients had N2b or greater at presentation. Similarly, comparatively few patients presented with T3 to T4 primary tumor in our study population (28% vs 44%). However, the age was similar (58.1 vs 57.0). The large percentage of local patients stratified as low risk by O’Sullivan et al.’s model may be an important consideration in model choice.

With the exception of N3, the distribution of nodal stage was similar between our local population and the population examined by Dahlstrom and colleagues. A similar trend held true for tumor staging. Gender and age were also highly similar. Therefore, the risk distribution patterns of our local population are in keeping with the patterns in the model population.

The mean age of our p16 + OSCC patients was similar to Huang’s (58.1 vs 57.8), as was the proportion of T1 – T3, N0 – N2c patients with a pack-year history greater than 20 pack-years [12]. Both the study population and the model are Canadian, and therefore it is expected the age and smoking history would be similar. The group considered to be of highest risk were those patients over the age of 70 with either a T4 or N3 tumor, of which our study identified no patients. In fact, the majority of discrepancy between the populations can be accounted for by the low prevalence of T4 or N3 tumors in our population. Patients with either T4 or N3 were twice as common in the model study population. Interestingly, both Huang et al. [12] and O’Sullivan et al. [13] examined populations in Ontario, Canada, and the cancer characteristics were more advanced in those populations. Additionally, the two largest single risk category differences occur in both of these models. It cannot be determined at this time if this is a result of geographical or institutional difference.

The strengths of this study are numerous. It is the first study to directly compare a local study population to multiple putative risk stratification models, two of which were created with similar geographical and jurisdictional populations. The local study population utilized a time period that encompasses the majority of time in which p16 immunohistochemistry was reliably completed at our institution. Multiple databases for identification of potential patients were utilized.

There were several limitations to this study. Despite representing a large portion of cases in our geographical area, the sample size was relatively small. Two databases were used in order to maximize the number of patients included in the study, including the provincial Cancer Care registry and an institutional transoral laser microsurgery database. Despite this consideration, it is impossible to determine if any cases were missed.

This study was not designed as an external validation of the compared risk stratification models, nor do our findings allow for determination if any of the examined models are applicable for determination of mortality in our local population. Revisiting the local patient population and validating the examined models with mortality data would be reasonable in the future.

In summary, this study examined the risk stratification distribution of the p16 + OSCC population in Nova Scotia. This was the first study to compare a local study population to multiple putative risk stratification models. This study also allowed for important demographic information to be gleaned about our local p16 + OSCC population, including smoking history, age, and sex distribution.

The majority of patients were found to be low risk by several putative risk stratification models. The hypothesis that the Nova Scotian p16 + OSCC population would have a lower proportion of low-risk patients was challenged. In concordance with the high rate of tobacco exposure in the Nova Scotia on the whole, the local p16 + OSCC population had a higher prevalence and quantity of tobacco exposure when compared to the populations from other jurisdictions used to validate the available risk stratification models.

## Abbreviations

AJCC: American Joint Committee on Cancer
HPV: Human papillomavirus
OSCC: Oropharyngeal squamous cell carcinoma
SCC: Squamous cell carcinoma

## References

[1] Canadian Cancer Society’s Advisory Committee on Cancer Statistics. Canadian Cancer Statistics 2016. Toronto, ON: Canadian Cancer Society; 2016.

[2] Blot WJ, McLaughlin JK, Winn DM (1988). Smoking and drinking in relation to oral and pharyngeal cancer. Cancer Res.

[3] Auluck A, Hislop G, Bajdik C, Poh C, Zhang L, Rosin M (2010). Trends in oropharyngeal and oral cavity cancer incidence of human papillomavirus (HPV)-related and HPV-unrelated sites in a multicultural population: the British Columbia experience. Cancer.

[4] Johnson-Obaseki S, McDonald JT, Corsten M, Rourke R (2012). Head and neck cancer in Canada: trends 1992 to 2007. Otolaryngol Head Neck Surg.

[5] Habbous S, Chu KP, Lau H (2017). Human papillomavirus in oropharyngeal cancer in Canada: analysis of 5 comprehensive cancer centres using multiple imputation. Can Med Assoc J.

[6] Nguyen-Tan PF, Zhang Q, Ang KK (2014). Randomized phase III trial to test accelerated versus standard fractionation in combination with concurrent cisplatin for head and neck carcinomas in the Radiation Therapy Oncology Group 0129 trial: long-term report of efficacy and toxicity. J Clin Oncol.

[7] Lee MK, Nalliah RP, Kim MK (2011). Prevalence and impact of complications on outcomes in patients hospitalized for oral and oropharyngeal cancer treatment. Oral Surg Oral Med Oral Pathol Oral Radiol Endod.

[8] Fakhry C, Westra WH, Li S (2008). Improved survival of patients with human papillomavirus–positive head and neck squamous cell carcinoma in a prospective clinical trial. J Natl Cancer Inst.

[9] Cmelak A, Li S, Marur S (2014). E1308: reduced-dose IMRT in human papilloma virus (HPV)-associated resectable oropharyngeal squamous carcinomas (OPSCC) after clinical complete response (cCR) to induction chemotherapy (IC). ASCO Annual Meeting Proceedings.

[10] Ang KK, Harris J, Wheeler R (2010). Human papillomavirus and survival of patients with oropharyngeal cancer. N Engl J Med.

[11] Dahlstrom KR, Garden AS, William WN, Lim MY, Sturgis EM (2016). Proposed staging system for patients with HPV-related oropharyngeal Cancer based on nasopharyngeal Cancer N categories. J Clin Oncol.

[12] Huang SH, Xu W, Waldron J (2015). Refining American joint committee on Cancer/Union for International Cancer Control TNM stage and prognostic groups for human papillomavirus-related oropharyngeal carcinomas. J Clin Oncol.

[13] O'Sullivan B, Huang SH, Siu LL (2013). Deintensification candidate subgroups in human papillomavirus-related oropharyngeal cancer according to minimal risk of distant metastasis. J Clin Oncol.

[14] Keane FK, Chen YH, Neville BA (2015). Changing prognostic significance of tumor stage and nodal stage in patients with squamous cell carcinoma of the oropharynx in the human papillomavirus era. Cancer.

[15] Lydiatt WM, Patel SG, O'Sullivan B (2017). Head and neck cancers—major changes in the American joint committee on cancer eighth edition cancer staging manual. CA Cancer J Clin.

[16] Statistics Canada . Focus on Geography Series, 2011 Census. In: products, Analytical , ed. Ottawa, Ontario: Statistics Canada Catalogue no. 98-310-XWE2011004; 2012.

[17] StatisticsCanada. Smokers, by sex, provinces, and territories. 2016. http://www.statcan.gc.ca/tables-tableaux/sum-som/l01/cst01/health74b-eng.htm2017). Accessed 10 Jan 2019.

[18] Nova Scotia Annual Statistical Report - Fiscal 2007/08. Nova Scotia Department of Health; 2008. p. 21.

[19] Jones A, Gulbis A, Baker EH (2010). Differences in tobacco use between Canada and the United States. Int J Public Health.

